# Genome-wide identification and characterization of replication origins by deep sequencing

**DOI:** 10.1186/gb-2012-13-4-r27

**Published:** 2012-04-24

**Authors:** Jia Xu, Yoshimi Yanagisawa, Alexander M Tsankov, Christopher Hart, Keita Aoki, Naveen Kommajosyula, Kathleen E Steinmann, James Bochicchio, Carsten Russ, Aviv Regev, Oliver J Rando, Chad Nusbaum, Hironori Niki, Patrice Milos, Zhiping Weng, Nicholas Rhind

**Affiliations:** 1 Bioinformatics and Integrative Biology, University of Massachusetts Medical School, Worcester, MA 01605, USA; 2 Bioinformatics Core Facility, University of Massachusetts Medical School, Worcester, MA 01605, USA; 3 Biochemistry and Molecular Pharmacology, University of Massachusetts Medical School, Worcester, MA 01605, USA; 4 Broad Institute of Massachusetts Institute of Technology and Harvard, Cambridge, MA 02142, USA; 5 Helicos BioSciences Corporation, Cambridge, MA 02139, USA; 6 Microbial Genetics Laboratory, Genetic Strains Research Center, National Institute of Genetics, 1111 Yata, Mishima, Shizuoka 411-8540, Japan; 7 Howard Hughes Medical Institute, 4000 Jones Bridge Road, Chevy Chase, MD 20815-6789, USA; 8 Division of Natural Sciences, New College of Florida, Sarasota, FL 34243, USA; 9 Broad Institute of Massachusetts Institute of Technology and Harvard, Cambridge, MA 02142, USA

## Abstract

**Background:**

DNA replication initiates at distinct origins in eukaryotic genomes, but the genomic features that define these sites are not well understood.

**Results:**

We have taken a combined experimental and bioinformatic approach to identify and characterize origins of replication in three distantly related fission yeasts: *Schizosaccharomyces pombe*, *Schizosaccharomyces octosporus *and *Schizosaccharomyces japonicus*. Using single-molecule deep sequencing to construct amplification-free high-resolution replication profiles, we located origins and identified sequence motifs that predict origin function. We then mapped nucleosome occupancy by deep sequencing of mononucleosomal DNA from the corresponding species, finding that origins tend to occupy nucleosome-depleted regions.

**Conclusions:**

The sequences that specify origins are evolutionarily plastic, with low complexity nucleosome-excluding sequences functioning in *S. pombe *and *S. octosporus*, and binding sites for trans-acting nucleosome-excluding proteins functioning in *S. japonicus*. Furthermore, chromosome-scale variation in replication timing is conserved independently of origin location and via a mechanism distinct from known heterochromatic effects on origin function. These results are consistent with a model in which origins are simply the nucleosome-depleted regions of the genome with the highest affinity for the origin recognition complex. This approach provides a general strategy for understanding the mechanisms that define DNA replication origins in eukaryotes.

## Background

*Cis*-acting sequences determine the location of replication origins in eukaryotic genomes [1]. However, the nature of such *cis-*acting sequences is not well understood. In the budding yeast *Saccharomyces cerevisiae*, the eukaryote with the best-studied origins, two sequence characteristics are important for origin function. The first is the 17-bp autonomously replicating sequence (ARS) consensus sequence (ACS) bound by the origin recognition complex (ORC) [2]. The second is a broader sequence context encompassing 200 to 300 bp that appears to be important for depleting nucleosomes from the origin [3-6]. The ACS is not sufficient for origin function; it is present in greater than ten-fold excess over functional origins [4,6]. Moreover, the ACS does not appear to be essential for ORC function *in vitro *[7]. Nonetheless, it is required for origin function *in vivo *[8]. Thus, it is thought that the ACS serves to increase the affinity of a subset of nucleosome-free regions (NFRs) for ORC, thereby conferring origin activity on these loci [4]. In other eukaryotes, the same characteristics - nucleosome depletion and ORC affinity - are likely to be important [9]. However, neither the mechanism by which nucleosomes are excluded nor the sequences that may bind ORC are known. In fact, the lack of obvious origin-specific motifs in other species and the lack of sequence-specific DNA binding of ORC *in vitro *has led to the speculation that any region of open chromatin in the genome permissive for ORC binding may function as an origin [4,10].

In the fission yeast *Schizosaccharomyces pombe*, origins have been mapped genome-wide by a number of approaches [11-15]. These maps confirm earlier conclusions that origin function in *S. pombe *is conferred by high AT content [16,17]. *S. pombe *ORC directly binds AT-rich sequences through nine AT-hook motifs at the amino terminus of its Orc4 subunit [18]. In addition, AT-rich sequences serve to exclude nucleosomes because the intrinsic stiffness of polyA energetically disfavors nucleosome formation [19,20]. NFRs at *S. pombe *origins have been both predicted and observed, although due to the low resolution of existing origin maps and consequent heterogeneity in origin alignments, the averaged NFRs are broader and shallower than those in *S. cerevisiae *[4,21,22].

The characterization of origin sequences in other organisms has been complicated by the difficulty of identifying origins at high resolution. ARS assays, which were essential in identifying yeast origins, have failed to identify origins in human cells [10]. Human origins that have been individually mapped at high resolution have not yielded predictive sequence motifs [23]. Recent identification of *Drosophila melanogaster *origins by ORC ChIP-chip showed that the sequences that determine origin function tend to be those that exclude nucleosomes; however, no origin-specific sequences were identified [9].

We have developed a generally applicable deep-sequencing-based approach to map origins at high resolution and characterize their sequence features. We applied our approach to three distantly related fission yeasts and found that although the sequences and genomic locations of origins are not conserved among the three species, sequence features that define origin function can be readily identified in each of the genomes. Although the sequence character of origins is conserved between *S. pombe *and *Schizosaccharomyces octosporus*, it has dramatically diverged in the evolution of *Schizosaccharomyces japonicus*.

## Results

### Identification of origins by deep sequencing

**Figure 1:**
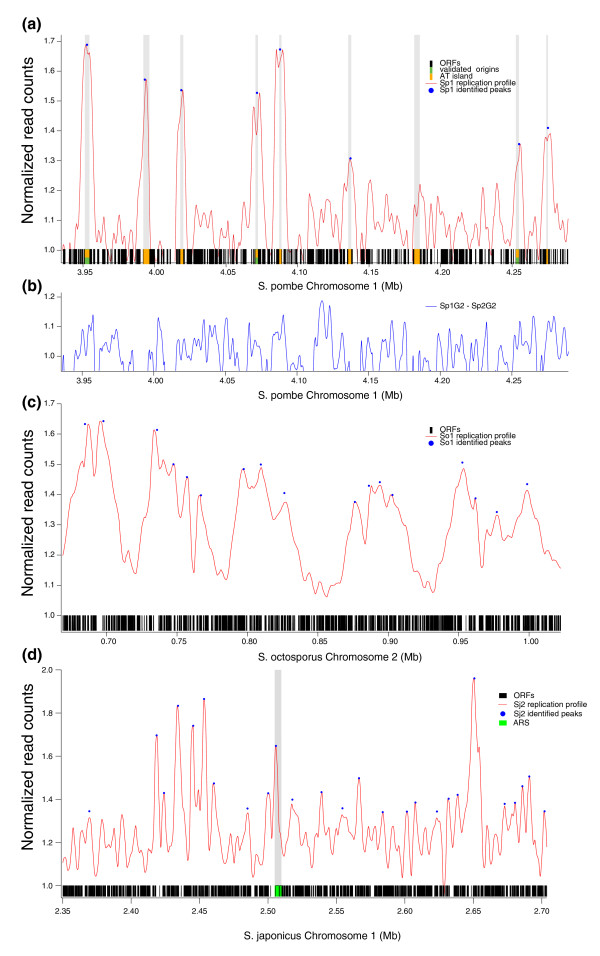
**Identification of replication origins by deep sequencing of hydroxyurea-arrested cells**. **(a) **The replication profile of a region of *S. pombe *dataset Sp1 containing three validated origins and several AT islands. The red curve shows the normalized replication fraction in HU-arrested cells. The blue dots show identified replication peaks. On the x-axis, black represents genes, orange represents AT-rich intergenes [17] and green represents validated origins (collated in [15]). **(b) **The difference between the G2 datasets form Sp1 and Sp2, demonstrating the magnitude of the noise in the datasets. **(c) **The replication profile of a region of *S. octosporus *dataset So1, as in (a). **(d) **The replication profile of a region of *S. japonicus *dataset Sj2 containing two cloned ARSs [28], as in (a). ORF, open reading frame.

To identify origins in *S. pombe*, *S. octosporus*, and *S. japonicus*, we mapped all sites with increased DNA copy number in early S phase [11,24]. We synchronized cells in G2 by elutriation, allowed them to enter S phase in the presence of the ribonucleotide reductase inhibitor hydroxyurea (HU), harvested genomic DNA from the S-phase arrested cells, sheared the DNA to about 200-bp fragments, and used deep sequencing to count the frequency of each region of the genome (Figure 1). As a control for systematic biases in DNA preparation and sequencing, we also sequenced DNA from G2 cells, which have uniform DNA copy number.

We employed Helicos single-molecule sequencing technology because it economically provides large numbers of reads without amplification and with significantly lower GC-bias than alternative sequencing technologies [25,26]. For *S. pombe*, we collected three pairs of datasets (each pair corresponding to S and G2 cells): two from wild-type cells with different synchronization protocols and one from a different strain background (see below). For *S. octosporus*, we collected one pair of wild-type datasets. For *S. japonicus*, we collected two wild-type S-phase datasets and one wild-type G2 dataset. For each sample, we collected an average of 18 million mappable sequence reads, which represents >200-fold sampling of copy number at a 200-bp resolution (Table 1).

**Table 1:** Dataset characterization

Name	Species	Genotype	Synchronization	S reads (M)	G2 reads (M)	Number of peaks	Median inter-peak distance	SVM auROC	Number of features used
Sp1	*S. pombe*	Wild type	Elutriation-HU	9.4	19.4	143	37.9	0.81	693
Sp2	*S. pombe*	*dpf1-3A*	Elutriation-HU	12.0	20.3	160	33.7	0.85	346
Sp3	*S. pombe*	*cdc25-ts*	*cdc25 *release-HU	9.7	7.7	387	18.2	0.76	1,386
So1	*S. octosporus*	Wild type	Elutriation-HU	37.5	31.3	208	22.1	0.77	346
Sj1	*S. japonicus*	Wild type	Elutriation-HU	10.3	24.5	526	14.2	0.86	1,386
Sj2	*S. japonicus*	Wild type	Elutriation-HU	9.6	24.5	542	13.8	0.89	693

To produce replication profiles for each species, we normalized the read frequency in the S and G2 datasets, subtracted the G2 background from the S-phase signal, and smoothed the resulting curves (see Materials and methods). The replication peaks of *S. pombe *and *S. japonicus *are about 10 kb wide, consistent with previous estimates of fork progression in HU arrested cells (Figure 1) [27]. The replication peaks of *S. octosporus *are more than twice as wide, perhaps reflecting a more heterogeneous arrest in HU.

We found that many of the peaks in the *S. pombe *Sp1 and Sp2 datasets were notched, presumably due to loss of bubble-shaped replication intermediates during our sample preparation. To compensate for the notched peaks, we identified peaks in all of our replication profiles using a template fitting approach that predicts where the peaks would be if they were not notched (Figure 1; Figure S1 in Additional file 1). To estimate the sensitivity of our peak detection, we compared two independent *S. pombe *G2 samples from the datasets Sp1 and Sp2 (Figure 1b). The difference between the two datasets, which reflects the experimental noise in the data, has a distribution of 1.00 ± 0.145. We therefore restricted our analysis to peaks of heights greater than 1.30, or about 2 standard deviations above the baseline. To confirm the reproducibility of our peaks, we compared independent datasets from both *S. pombe *and *S. japonicus *and found a strong correlation between peak locations in the datasets (*P *< 10^-10^; Figure S2 in Additional file 1).

To validate our replication peaks, we compared our data to confirmed origins. For *S. pombe*, we examined 37 origins that were previously shown to be active *in vivo *by two-dimensional gel analysis (collated in [15]) and have mappable reads in our datasets. Of the 37 origins, 22 overlap with an identified replication peak in our data sets; 8 are less than 1 kb away and 4 are between 1 and 2.5 kb away; for 3 we see no replication peak in the region (Figure 1a; Table S2 in Additional file 2). Overall, the enrichment of origins near replication peaks is highly significant (*P *= 4.41 × 10^-09^). We also compared our replication peaks to AT islands, intergenes of high AT content, which are strong predictors of origin function in *S. pombe *[17]. We find a significant (*P *< 10^-10^), but not perfect, overlap of AT islands with our replication peaks (Figure 1a), consistent with our sequence analysis of origin motifs (see below). We also see comparable overlap between our replication peaks and published *S. pombe *origin maps (see Materials and methods).

For *S. japonicus*, we compared our data to 11 ARSs isolated from a genomic library [28]. Six of the ARSs encompass a replication peak and four others were within 2.5 kb of a replication peak (*P *= 0.012); for one ARS, we found no associated replication peak (Figure 1d; Table S3 in Additional file 2). These results suggest that our replication peaks correspond to *in vivo *origins of replication.

### Genome scale origin distribution

We compared the locations of replication peaks within syntenic regions among the three species and found no significant conservation of origin location (*P *= 0.22), consistent with the lack of conservation of origin locations among budding yeast species of similar evolutionary distance [29].

**Figure 2:**
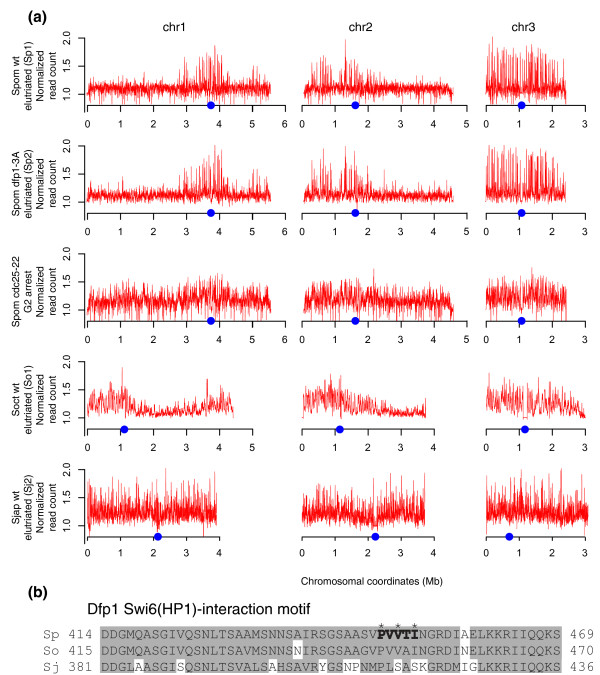
**Genome-wide patterns of origin firing**. **(a) **The extent of replication in HU-arrested cells is depicted in red. The locations of centromeres are indicated by blue dots. **(b) **The Swi6(HP1)-binding region of Dfp1 from *S. pombe*, *S. octosporus *and *S. japonicus*. The *S. pombe *Swi6-binding motif is in bold. The three amino acids mutated in *dfp1-3A *are indicated by asterisks. Amino acids similar in two of the three species are boxed in gray. The presence of the *dfp1-3A *allele inHM1826 was confirmed from the genomic sequence used to generate the replication profiles. Wt, wild type.

Nonetheless, genome-scale patterns of origin firing are conserved between *S. pombe *and *S. octosporus *(Figure 2). The replication peaks on chromosomes 1 and 2 of these species are greatly enriched in the central, centromere-containing regions of the chromosomes and much diminished on the arms; in contrast, replication peaks are uniformly distributed across chromosome 3. Early firing of *S. pombe *centromeric origins has been reported [30]. This early firing is due to an interaction between the Dfp1-dependent kinase (DDK; the *S. pombe *homolog of the Cdc7-Dbf4 replication kinase) with the Swi6 heterochromatin-binding protein (the *S. pombe *homolog of the HP1 heterochromatin-binding protein) [31]. The Swi6-binding motif in Dfp1 is conserved in *S. octosporus*, which also shows a bias towards chromosome centers, but not in *S. japonicus*, which does not (Figure 2b). To test if the Dpf1-Swi6 interaction is required for the centromere bias, we mapped origins in an *S. pombe *strain carrying a mutant Dfp1 that does not interact with Swi6 [31]. The mutant does not affect the centromere bias (Figure 2a), consistent with the observation that the bias extends hundreds of kilobases from the centromeres, much farther than heterochromatin does, and encompasses all of chromosome 3.

The central regions of chromosomes 1 and 2 have been previously reported to have more efficient origins than their arms [11,13,15], but the effect is not as strong as the bias seen in our datasets. We investigated experimental factors that might account for this difference in the distribution of origin firing. Previous experiments had synchronized cells in G2 by inactivating a temperature-sensitive (ts) allele of the Cdc25 mitotic activator and then releasing the blocked culture into a synchronous cell cycle. This protocol arrests cells for up to 4 hours in G2 and causes them to grow to as much as twice their normal size before dividing. In contrast, we had used centrifugal elutriation to select a synchronous population of G2 cells from an asynchronous culture, a protocol that does not involve arresting the cell cycle. We repeated our origin mapping using a *cdc25-ts *block-and-release approach, and confirmed that origin firing is much more uniformly distributed across the genome (Figure 2). Nonetheless, the same origins are used in the two conditions (Figure S2 in Additional file 1). This result suggests that a prolonged G2 arrest causes origin firing potential to be more uniformly distributed.

### Characterization of origin sequences

Although origin locations have been identified in a wide variety of eukaryotes, the sequence characteristics that confer origin activity to these loci have only been elucidated in *S. cerevisiae *and *S. pombe *[1]. We used two computational approaches to analyze the sequence characteristics of the origins we identified in the three *Schizosaccharomyces *species. The first approach used a support vector machine (SVM), a machine-learning algorithm that tests the ability of *k*-mer sequences (*k *= 1 to 6) to predict origin function (see Materials and methods) [32]. The results of such tests can be analyzed with a receiver operating characteristic (ROC) curve, which plots true-positive rate (sensitivity) as a function of false-positive rate (1 minus specificity). The area under the ROC curve (auROC) demonstrates the performance of the SVM model, with 1.0 being perfect and 0.5 being no better than random. auROC thus serves as a metric of the predictive power of the model.

**Figure 3:**
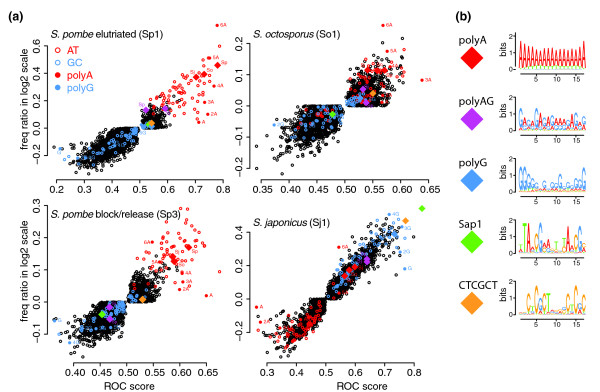
**Sequence characteristics of fission yeast origins**. **(a) **Each *k*-mer (*k *= 1 to 6) is plotted on origin prediction (auROC) versus origin enrichment (log frequency ratio). *k*-mers of only A or T are red and those of only G or C blue; polyA *k*-mers are solid red and polyG *k*-mers blue and labeled. Complementary sequences are not distinguished (that is, 6A is equivalent to 6T). Motifs identified by MEME are represented as diamond symbols. For MEME motifs identified in more than one species, the species of origin is indicated. **(b) **Logos of the MEME motifs included in (a). For MEME motifs identified in more that one species, a representative logo is shown.

**Figure 4:**
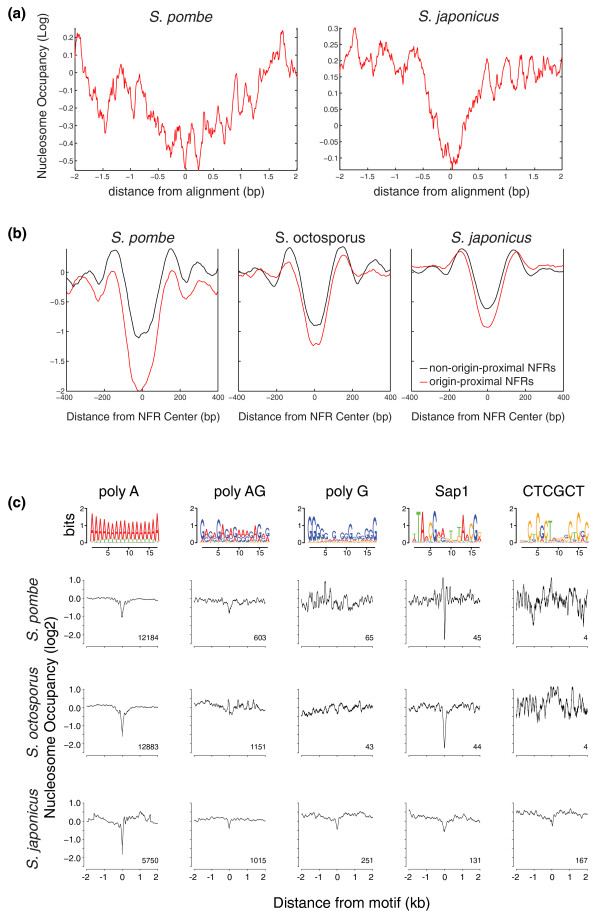
**Fission yeast origins are depleted of nucleosomes**. **(a) **Average nucleosome occupancy for all replication peaks relative to the genome average is plotted log_2_. **(b) **Average nucleosome occupancy of the NFRs nearest to each replication peak is plotted in red; the nucleosome occupancy of the NFRs nearest to an equal number of random non-origin locus is plotted in black. **(c) **Average nucleosome occupancy over all instances of each of the identified origin motifs relative to the genome average is plotted log_2_. The logo for each motif is shown above.

We performed SVM analysis on all data sets (Figure 3 and Table 1; Tables S4 and S5 in Additional file 2). For all three species, we are able to predict origins with high sensitivity and specificity, as demonstrated by auROC scores ranging from 0.76 to 0.89 (Table 1). To determine which sequences are the most predictive of origin function, we examined the auROC scores for individual *k*-mers. As expected for *S. pombe*, the AT richness is a strong predictor of origin function, as demonstrated by the high predictive value of the A nucleotide (auROC = 0.62 to 0.79; Table S4 in Additional file 2). However, simple AT richness is not as strong a predictor as polyA tracts (auROC up to 0.86), consistent with the fact that the AT-hook motif, of which *S. pombe *Orc4 has nine (Figure S4 in Additional file 1), binds polyA. Interestingly, the sequence character of the origins used in the *cdc25-ts*-synchronized culture is less biased towards strictly polyA and is less predictive of origin function than that of the unarrested, wild-type culture (Table 1; Table S4 in Additional file 2). This change in the sequence character of the origins used in the *cdc25-ts*-synchronized cells is consistent with a wider range of less well-defined origins being used in these experimental conditions. *S. octosporus *origins have similar sequence character, with polyA tracts dominating the SVM signal; however, the auROC scores are significantly lower than those for *S. pombe*, possible due to the lower resolution of the data set (Figures 2 and Figure 4a, Table 1; Table S4 in Additional file 2).

Surprisingly, AT content is not a positive predictor of origin function in *S. japonicus*. In fact, it is a strong negative predictor of origin function (auROC = 0.15). Instead, *S. japonicus *origins are characterized by high GC content and a variety of GC-rich *k*-mers. Interestingly, A is a much stronger negative predictor of origin function than 6A, which is a weakly positive character (auROC = 0.54). This result suggests that although *S. japonicus *origins are depleted in AT content, they maintain a higher relative polyA content, consistent with the existence of AT-hooks on *S. japonicus *Orc2 and Orc4 (Figure S4 in Additional file 1).

As expected, SMVs trained on one data set from a species performed well on other datasets from that species (Table S5 in Additional file 2). Conversely, the SMVs trained on *S. pombe *perform poorly on *S. japonicus*, which has very different origin characteristics, and vice versa. Interestingly, the *S. pombe *SVMs also performed poorly on *S. octosporus*, and vice versa. Therefore, it appears that even though *S. pombe *and *S. octosporus *have qualitatively similar origin characteristics, there are quantitative differences in their origin sequences.

Our second approach to characterizing origin sequence used the *ab initio *motif discover algorithm MEME, which is capable of identifying the enrichment of motifs that are longer than six residues. As for the SVM analysis, we used 1 kb from either side of the replication peaks as the positive loci. However, because fission yeast origins are generally intergenic [16,17], our positive loci are enriched for promoter sequences. To avoid training on promoter sequences and thus inadvertently identifying promoter motifs, we masked coding sequence from both our positive and negative loci. We performed the analysis using two background models. The first approach uses only the mono-nucleotide composition of the input loci as background, and finds motifs that are enriched relative to that bias. This approach finds motifs that are unexpectedly frequent in the input loci, but does not compare the frequency between positive and negative loci. The second approach uses a fifth-order hidden Markov model of the background in the negative loci, which reduces the background noise and allows for more rigorous identification of enriched motifs. We initially limited our search to motifs of less than 17 bases, to increase sensitivity. No additional motifs were found upon extending the search space to 50 bases.

In both *S. pombe *and *S. octosporus*, the motifs identified by MEME using both background models reflected the polyA-rich nature of origins seen in the SVM analysis (Figure 3; Table S6 in Additional file 2). In addition, a polyAG motif was frequently found in *S. pombe *origins (Table S6 in Additional file 2).

In *S. japonicus*, using the mono-nucleotide composition background, we find polyA motifs, suggesting that origins have significant enrichment of polyA tracts above that expected from their AT content, consistent with the SVM results (Figure 3; Table S4 in Additional file 2). However, using the hidden Markov model background model, we find no polyA motifs, suggesting that although intergenes in general tend to have high numbers of polyA tracts, those tracts do not directly determine origin function. Instead, we find three highly enriched motifs (Figure 3): polyG, a head to tail repeat of CTCGCT and the binding site for the Sap1 protein (Figure S3 in Additional file 1), with 87% of all replication peaks containing at least one of the motifs. polyG motifs are known to exclude nucleosomes - like polyA, due to the intrinsic stiffness of the sequence [19,20,33] - and Sap1 has been shown to exclude nucleosomes in *S. pombe *[33], suggesting that these motifs may function to deplete origins of nucleosomes in *S. japonicus*.

### Nucleosome localization at origins

Previous work has demonstrated that origins tend to be nucleosome depleted [3,4,9,21,34]. To investigate the nucleosome occupancy of origins in the three fission yeast, we mapped mono-nucleosomes by deep sequencing of all three species. Average nucleosome occupancy profiles centered on replication peaks from *S. pombe *and *S. japonicus *show them to be nucleosome depleted, as expected (Figure 4a). The replication peaks for *S. octosporus *show no significant nucleosome depletion (data not shown), possibly due to the lower resolution of that replication data set. To compensate for the fact that our replication peaks are not perfectly aligned with their underlying origins, we identified the nearest NFR to each replication peak and to an equal number of control sites (Figure 4b). Average nucleosome occupancy profiles centered on origin-proximal NFRs tend to be deeper and broader than nucleosome profiles centered on origin-distal NFRs (*P *< 5 × 10^-3^). Note that both the origin NFRs and non-origin NFRs in *S. pombe *tend to be more nucleosome depleted than in *S. japonicus*. In *S. octosporus*, the non-origin NFRs are similar to those in *S. pombe*, but the *S. octosporus *origin NFRs appear to be less depleted than the origin NFRs in *S. pombe*, possibly because the lower resolution of the data set caused some of the replication peaks to be assigned to the wrong NFR.

Several of the identified sequence motifs associated with origins, in particular polyA and Sap1, are known to exclude histone binding [20,33]. To investigate the roles of other origin-associated motifs, we examined their *in vivo *nucleosome occupancy. The motifs tend to be nucleosome depleted in all three species, with three exceptions (Figure 4c). The first exception is the CTCGCT motif in *S. pombe *and *S. octosporus*, which is largely absent from these genomes. The second exception is polyG, which is not nucleosome depleted in *S. pombe *and *S. octosporus*. Although it seems counter-intuitive that a sequence believed to exclude nucleosomes because of its intrinsic stiffness should show species-specific effects, such effects have been seen before, due to the fact that polyG motifs in relatively G-rich genomes (like *S. japonicus*) tend to be embedded in longer G-rich sequences [33]. The third exception is the lack of nucleosome exclusion for polyAG in *S. octosporus*. This result is consistent with the fact the polyAG is not enriched at *S. octosporus *origins and may reflect a different dinucleotide bias between the *S. pombe *and *S. octosporus *polyAG sequences.

To further investigate the roles of the identified origin motifs in nucleosome exclusion, we compared the nucleosome occupancy of motifs at origins and non-origin loci. The motifs all tend to be as nucleosome depleted at non-origin loci as they are at origins (Figure S5 in Additional file 1). This result suggests that the motifs exclude nucleosomes, as opposed to being nucleosome depleted because of their enrichment in origins. Therefore, the sequences that characterize origins in fission yeast appear to fulfill two roles: excluding nucleosomes and binding ORC. This conclusion is consistent with a general model for eukaryotic origins in which origins are simply the NFRs with the highest affinity ORC binding sites.

## Discussion

We have used a combination of experimental and bioinformatic approaches to identify and characterize replication origins in three distantly related fission yeast. We find that origins are plastic genetic elements, lacking conservation of primary sequence or location. Nonetheless, origins maintain conserved sequence characteristics that reflect the need for two origin functions: exclusion of nucleosomes and binding of ORC. Moreover, we have identified sequence determinants that predict origin function. Surprisingly, the sequences predictive of origin function are qualitatively different between *S. pombe *and *S. japonicus*, suggesting that not only are specific origins evolutionarily ephemeral but that the mechanisms that define origins are not strongly constrained.

Analysis of origin locations relative to conserved genes in the three species show that origins are not conserved. Origin locations are conserved amongst the much more closely related budding yeast of the *Saccharomyces *sensu stricto clade [6]. However, even in that study, origin heterogeneity was observed. Origin locations are not conserved between *Saccharomyces cerevisiae *and *Kluyveromyces lactis *[29], which are less closely related (54.8% average amino acid identity) than *S. pombe *and *S. octosporus *(65.5% average amino acid identity) [35]. In contrast to the rapid divergence of origins within the *Schizosaccharomyces *clade, coding gene order and structure are strongly conserved; the median block of conserved synteny between *S. pombe *and *S. octosporus *is 65 kb (43 genes) and 81% of genes in the clade have conserved exon structure [35]. These results suggest that origins are dynamic genetic elements, any one of which is under weak evolutionary constraint.

In contrast to the lack of conservation of specific origins, the chromosome-wide pattern of origin firing is conserved between *S. pombe *and *S. octosporus*. In both species, early firing origins are concentrated in the central regions of the two larger chromosomes but spread across the smaller third chromosome (Figure 2). However, because of the numerous chromosomal rearrangements between the two species [35], this conserved chromosomal pattern does not imply any conservation of replication timing between the genes of the two species. *S. pombe *centromeres replicate early [30,31]; however, the early replicating regions seen in our data extend hundreds of kilobases beyond the centromeric heterochromatin (Figure 2). Furthermore, the interaction between the DDK replication kinase and centromeric heterochromatin required for the early replication of *S. pombe *centromeres [31] is not required for the chromosome-wide pattern of origin firing we see in *S. pombe *(Figure 2). These results suggest that the distribution of origin firing is regulated by factors other than centromeric heterochromatin.

The difference in origin-firing distribution along chromosomes is reduced in magnitude when cells are arrested in G2 before replication (Figure 2). A similar effect is seen when cells are arrested in metaphase [36]. Since both G2 and metaphase arrests lead to a similar redistribution of origin-firing timing, we suspect that the effect is not due to the specific phase of the cell-cycle arrest. In fact, since origins are not licensed in G2, we suspect it is not a direct effect on origins at all. Instead, we speculate that it is the increased size of the cells during the arrest that affects origin firing. Origin efficiency in *S. pombe *is regulated by the rate limiting factors Dfp1 (the regulatory subunit of the DDK replication kinase) and Cdc45 (an origin initiation factor) [36,37]. Since cells continue to grow while arrested, these proteins will continue to accumulate. Upon release, the excess of these limiting factors may allow normally less efficient origins to compete more efficiently for firing. A similar situation is observed in human cells, in which synchronization by release from G1 or S block allows for the activation of normally inefficient or dormant origins [38].

The sequences that predict origin function in the *Schizosaccharomyces *clade appear to have two functions: ORC binding and nucleosome exclusion. All three species have AT hook domains on their Orc4 subunits (Figure S4 in Additional file 1), which, in *S. pombe*, direct ORC binding to polyA sequences [18]. As expected, origins in all three species are enriched for polyA motifs (Figure 3; Tables S4 and S5 in Additional file 2). In *S. pombe *and *S. octosporus*, origins are defined by polyA motifs [16,17], which not only bind ORC but also intrinsically exclude nucleosomes, due to the biophysical stiffness of polyA sequence [20]. The lack of specific origin motifs in *S. pombe *is consistent with the finding that synthetic A-rich sequences confer origin function [39]. In contrast, in *S. japonicus*, polyA is not predictive of origin function, suggesting it acts like the ACS in *S. cerevisiae*, which is required to bind ORC, but only functions in an NFR context [4,6]. It is interesting to note that *S. japonicus *has a single amino-terminal AT hook on Orc2, a configuration shared with *S. cerevisiae*, but not with the other fission yeast. This evolutionary distribution suggests that the Orc2 AT hook is ancestral and was lost in the *S. pombe*-*S. octosporus *clade. Likewise, the presence of Orc4 AT hooks in *S. japonicus *suggests that these motifs arose before AT motifs evolved to define origins, allowing evolution of AT-rich origins and the loss of other origin defining motifs in the *S. pombe*-*S. octosporus *clade.

Instead of using polyA sequences to exclude nucleosomes, *S. japonicus *origins are defined by other nucleosome-excluding sequences (Figure 3): polyG, the Sap1 binding site and a CTCGTC motif. polyG motifs exclude nucleosomes, presumably due to the intrinsic stiffness of the sequence [19,33]. polyG motifs are nucleosome depleted in other yeast [33], but not in *S. pombe *or *S. octosporus *(Figure 4c). This discrepancy is likely due to the *S. japonicus *polyG motifs being embedded in larger G-rich stretches, an effect seen within the budding yeast clade [33]. polyG is also enriched in human origins [40] and is anti-nucleosomal in *C. elegans *[33], suggesting that polyG sequences may have a general role in excluding nucleosomes from origins in relatively G-rich metazoan genomes.

Sap1 is a *trans*-acting nucleosome-excluding factor [33] with binding sites enriched at *S. japonicus *origins (Figure 3; Table S6 in Additional file 2). The fact that Sap1 is strongly anti-nucleosomal in *S. pombe *and *S. octosporus *[33] (Figure 4c; Figure S5 in Additional file 1) but not enriched in *S. pombe *or *S. octosporus *origins (Figure 3a) suggests that Sap1 is a general nucleosome excluding factor, consistent with its roles in a number of other functions in *S. pombe *[41-43]. Sap1 sites are found primarily in origins in *S. japonicus *(113/131, 86%), but not in *S. pombe *or *S. octosporus *(12/45, 27%). Interestingly, one of Sap1's essential functions in *S. pombe *involves Cbf1 [41], a protein specific to the *S. pombe*-*S. octosporus *clade [35], suggesting that Sap1 may have evolved new functions in the *S. pombe*-*S. octosporus *clade. However, Sap1 still retains a role in replication in *S. pombe *[42], even if it no longer defines replication origins.

The CTCGCT motif is also likely to act by binding a *trans*-acting factor. The fact that the motif is essentially absent from the *S. pombe*-*S. octosporus *clade suggests that it is bound by an *S. japonicus*-specific nucleosome-excluding factor.

## Conclusions

Our comparative analysis of origins in fission yeast has allowed us to identify the sequence characteristics that define origin function in these species. Our results are consistent with a general model for eukaryotic origin function in which origins are simply the nucleosome-free regions in the genome with the highest affinity for ORC [4]. In metazoans, where ORC appears to have little, if any, sequence specificity [1], this model suggests that any NFR could act as an origin, consistent with the distributed initiations seen at some loci [44] and the correlation between origins and promoters [9,40], which are also nucleosome free. Furthermore, the model suggests that origins are not well-defined genetic elements, but simply the highest affinity ORC binding sites available in the genome. As long as there are no large regions of the genome devoid of ORC binding sites, the particular location or characteristics of these sites may not be important.

## Materials and methods

### General methods

All three species were grown in rich medium (YES) and manipulated using standard fission yeast protocols [45]. The strains used were yFS101 (*S. pombe h-*), HM1826 (*S. pombe h+ nmt1-TK dfp1-3A::kanMX6*) [31], yFS128 (*S. pombe h- leu1-32 ura4-D18 cdc25-22*), yFS286 (*S. octosporus*), and yFS275 (*S. japonicus*).

### DNA sequencing

DNA sequencing and sequence data analysis were performed as previously described [26]. We used the January 2007 assembly of the *S. pombe *genome [46] and SO3 and SJ1 assemblies of the *S. octosporus *and *S. japonicus *genomes [35]. Reads that mapped to multiple locations of the genome were discarded. Post analysis, *S. japonicus *coordinates were mapped to genome assembly SJ4, the current release.

### Construction of genome-wide replication profiles

To construct replication profiles, we extended the reads by 100 bp in the 3' direction, because the average length of DNA fragments was 200 bp. Then we smoothed the data using a 200-bp sliding window with a 20-bp step. We excluded regions of *S. octosporus *(contig6:1-400000, contig2:1-5600, contig7:766887-886887) and *S. japonicus *(contig1:1-62000, contig3:1-74000, contig3:1776000-1809320, contig4:1-16000) that had anomalously high read counts, presumably due to under-representation of repetitive sequences in the genome assemblies. To account for different sequencing depths in the various datasets, we normalized each dataset so that the mean across the genome was 1. Using flow cytometry analysis of percentage of the genome replicated in the S phase samples (Table S1 in Additional file 2), we applied the following formula to combine the S and G2 datasets into replication profiles: *R *= *S *× (1 + *n*%) - *G2 *+ 1. Finally, we performed LOESS smoothing (Igor PRO v6.12, WaveMetrics (Lake Oswego, OR USA) on the replication profile *R *using empirically determined smoothing factors (Table S1 in Additional file 2).

### Replication origin peak detection

We used the MATLAB ipeak program (MathWorks (Natick, MA USA) to detect peaks in each replication profile *R*. The ipeak algorithm first looks for downward zero crossings in smoothed first derivative, and then detects peaks by least square curve fitting. The ipeak parameters used were AmpT = 1, SlopeT = 0, SmoothW = 58, FitW = 58 for *S. pombe*, AmpT = 1, SlopeT = 0, SmoothW = 50, FitW = 50 for *S. octosporus*, and AmpT = 1, SlopeT = 0, SmoothW = 10, FitW = 10 for *S. japonicus*. We set the peaks to be the highest positions in *R *within a ± 500-bp window around the peak locations detected by ipeak. Peaks with anomalous heights (greater than 3 in *S*. *pombe *and *S. octosporus *or 10 in *S*. *japonicus*) were discarded; these regions are presumed to correspond to unannotated repetitive regions. We used the Genomic Signal Aggregator [47] to generate an average peak profile of all peaks detected by ipeak in the entire genome. The center of the average peak profile was then fitted to a Gaussian distribution, which was then used serve as a peak template for refining peak calling.

In order to remove split peaks and sub-peaks, we used the following strategy. First we sorted all peaks detected by the ipeak algorithm by their amplitudes. Then we went down the list and performed the following steps: 1) we scaled the peak template to the same height as the data peak under investigation and positioned the template such that its highest point coincides with the highest point of the data peak; 2) we slid the peak template in a ± 250-bp window to search for the position that would lead to maximal correlation between the peak template and the data peak; 3) we searched in the height dimension with a step of 0.01 for minimal root mean square deviation (RMSD) between the peak template and the data peak. To avoid noise in the data, the correlation and RMSD were computed only for the region that was within 60% of the highest point of the data peak. We assigned the peak location and height to those of the resulting template, saved the information, then subtracted the newly detected peak from the replication profile *R *so that minor side peaks would be ignored. We iterated this procedure until all peaks above the peak threshold had been detected.

### Validation of origin peak detection

We compared our data to published *S. pombe *origin lists compiled by three different approaches: MCM ChIP-chip mapping, a microarray-based, HU-arrest, copy-number strategy similar to ours, and a microarray-based mapping of single-stranded DNA in HU arrested cells [11-13]. We calculated the statistical significance of the overlap between these origin lists (the test sets) and two well-defined origin lists (the validation sets): a list of origins confirmed by two-dimensional gels (collated in [15]) and a list of AT-rich intergenes that are strongly correlated with origins [17]. We varied the resolution for the test sets, from 500 to 5,000 bp, by varying the distance from a validation set origin that a test set origin could be and still be considered to overlap it. We reasoned that the statistical significance of the overlap between the test and validation sets would increase as the size of the test sets increased up to the resolution of the test set. After that point the significance would decrease because the test set would get bigger without improving the overlap. By this analysis, our data are of similar resolution, between about 1 and 3 kb, to the other HU-arrest approaches (see Materials and methods; Table S7 in Additional file 2), which presumably reflects the resolution of the HU-arrested replication bubbles. The overlap between our replication peaks is comparable to previously published origin prediction, with approximately 50% overlap between any pairwise comparisons of datasets (Figure S2 in Additional file 1).

### Motif finding

We used MEME v4.4 to find sequence motifs *de novo *in the replication origin peaks with parameters nmotifs = 30, minsites= 20, minw = 11, maxw = 17 [48]. MEME is an expectation-maximization algorithm that identifies motifs that are enriched in a positive set of sequences, in our case the sequences from 1 kb on either side of the replication peaks, compared with a background set of sequences, which were a set of sequences from 1 kb on either side of loci chosen randomly from the genome excluding 8 kb around the peaks taller than 1.1, 1.14, 1.18, 1.2, 1.2, and 1.25 for Sp1, Sp2, Sp3, So1, Sj1, and Sj2, respectively. We ran the algorithm with two background models: fifth-order Markov model (defined as frequencies of all 6-mers) or simply mononucleotide frequencies. If two neighboring peaks were closer than 2 kb, the overlapping sequence was counted only once. We only used the intergenic portions for both positive and background sequences.

### SVM peak prediction

In order to predict replication origins from sequence features, we constructed SVMs with a linear kernel using the libSVM algorithm in the R package (e1071). We used *k*-mer (*k *= 1 to 6; 2,772 *k*-mers in total) and significant motifs identified by MEME as sequence features. The frequency of each *k*-mer was calculated for each sequence in the peak set and the background set as described in the Motif Finding section, and was 2-norm normalized. For each MEME motif, we ran the CLOVER algorithm to obtain a cumulative score for each sequence [49]. Then we normalized the CLOVER scores for each motif to have the same mean and standard deviation as the *k*-mer frequencies across the training sequences, so that *k*-mers and motifs could be combined with the SVM.

We used the positive and background sequences described in the Motif Finding section for training and testing the SVMs. We performed five-fold cross-validation, in which the sequences were divided into three equal sized portions and in each of five runs one portion is held out for testing while the other two portions were used for training. We computed the auROC score, which is a performance measure that combines sensitivity and specificity, using libSVM and colAUC (R package caTools).

### Nucleosome mapping

Nucleosomal DNA libraries were constructed and sequenced as previously described [50]. To assess the quality of the nucleosome maps, we aligned our nucleosome signal on the annotated transcriptional start site for each genome [35] using the Genomic Signal Aggregator [47] (Figure S6 in Additional file 1). The profiles show a deep NFR at the transcriptional start site, with phased nucleosomes downstream and heterogeneous nucleosomes upstream, as previously reported for *S. pombe *[21,33]. Nucleosome mapping and motif occupancy analysis were performed as previously described, using default settings [51]. We identified NFRs genome-wide as described [50] but without the constraint of searching relative to the translational start site of each gene. We defined the NFRs nearest each species' replication peaks as the origin-proximal NFRs and computed their average nucleosome occupancy, aligning the data on the NFR centers. As controls, we chose an equal number of randomly distributed genomic coordinates and defined the nearest NFRs as non-origin-proximal and then measured the non-origin-proximal average nucleosome occupancy. We repeated this analysis for 10,000 randomly chosen genomic regions for each species. To test the significance of the hypothesis that origin-proximal NFRs are more nucleosome depleted than non-origin-proximal NFRs, we calculated a *P*-value of n/10,000, where n is the number of times that the non-origin-proximal NFR had an average nucleosome occupancy that was more depleted than the origin-proximal NFRs.

### Data availability

Filtered reads for all datasets have been submitted to the Sequence Read Archive (Table S8 in Additional file 2). *S. pombe *replication profiles have been submitted to OriDB [52] and ReplicationDomain [53].

## Abbreviations

ACS: ARS consensus sequence; ARS: autonomously replicating sequence; auROC: area under the ROC curve; bp: base pair; DDK: Dbf4-depenent kinase; HU: hydroxyurea; NFR: nucleosome-free region; OCR: origin recognition complex; ROC: receiver operating characteristic; SVM: support vector machine; ts: temperature sensitive.

## Competing interests

CH, KS and PM were employees of Helicos BioSciences during the tenure of this research.

## Authors' contributions

JX performed most post-sequence-alignment data analysis. YY constructed nucleosomal DNA libraries. AT analyzed nucleosome occupancy. CH filtered and aligned the replication-profile sequence data. KA and HN isolated and characterized the *S. japonicus *ARSs. NK prepared the replication-profile DNA samples. KES prepared and sequenced the replication-profile DNA libraries. JB and CR provided additional DNA sequence and analysis. OJR analyzed nucleosome occupancy and interpreted data. ZW designed and facilitated data analysis. PM, CN and NR conceived the study and coordinated its execution. NR interpreted the data and wrote the manuscript. All authors read and approved the final manuscript.

## Supplementary Material

Additional file 1 **Supplemental Figures S1 to S6**. Figure S1: Peak detection by template fitting. Examples of the template-fitting approach to peak detection. The blue curve is the normalized, smoothed data. The blue dot is the peak call before template fitting. The red curve is the template. The red dot is the peak call after template fitting. The numbers are serial numbers assigned to the peaks during the iterative peak-calling process. Peak 106 can be seen at about 4.27 Mb in Figure 1 and Figure S2 in Additional file 1. Figure S2: Comparison of independent replication profiles. Replication profiles from **(a) **Sp1, Sp2 and Sp3 and **(b) **Sj1 and Sj2 are compared as in Figure 2. **(c) **Venn diagrams of peak overlap between the indicated datasets. Most cases of non-overlapping peak calls are due to the peak in one of the datasets being below the cutoff, such as at 4.23 Mb is *S. pombe *and 1.55 Mb is *S. japonicus*. Figure S3: The Sap1 binding site. Logos for the Sap1 binding site derived from **(a) **MEME analysis of *S. japonicus *origins (Figure 3; Table S6 in Additional file 2) and **(b) ***in vitro *selection [54]. Figure S4: Orc2 and Orc4 domain structures. Domain structures of Orc2 and Orc4 as defined by PFAM [55]. Figure S5: Origin motifs are nucleosome depleted are origin and non-origin sites. Nucleosome occupancy over motifs is depicted as in Figure 4, except motifs are divided into those within 1 kb of a replication peak and those farther away. Figure S6: Nucleosome alignment on transcriptional start sites. Nucleosome occupancy over all annotated transcriptional start sites is depicted. Click here for file

Additional file 2 **Supplemental Tables S1 to S8**. Table S1: dataset analysis parameters. Table S2: validated *S. pombe *origins. Table S3: *S. japonicus *ARSs. Table S4: *k*-mer auROC scores. Table S5: cross-species SVM performance. Table S6: MEME results. Table S7: dataset overlap. Table S8: accession numbers. Click here for file
