# Supplementary figures and images for: Genome-wide identification and characterization of replication origins by deep sequencing
Source: Genome Biol. 2012 Apr 24;13(4):R27. doi: 10.1186/gb-2012-13-4-r27 (PMC3446301; doi:10.1186/gb-2012-13-4-r27)

Figure S1

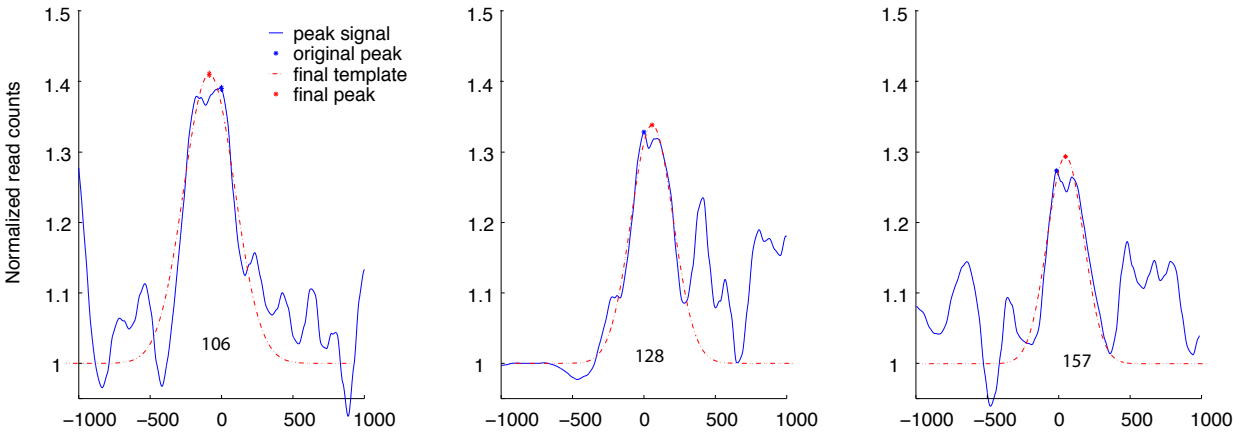

Figure S2

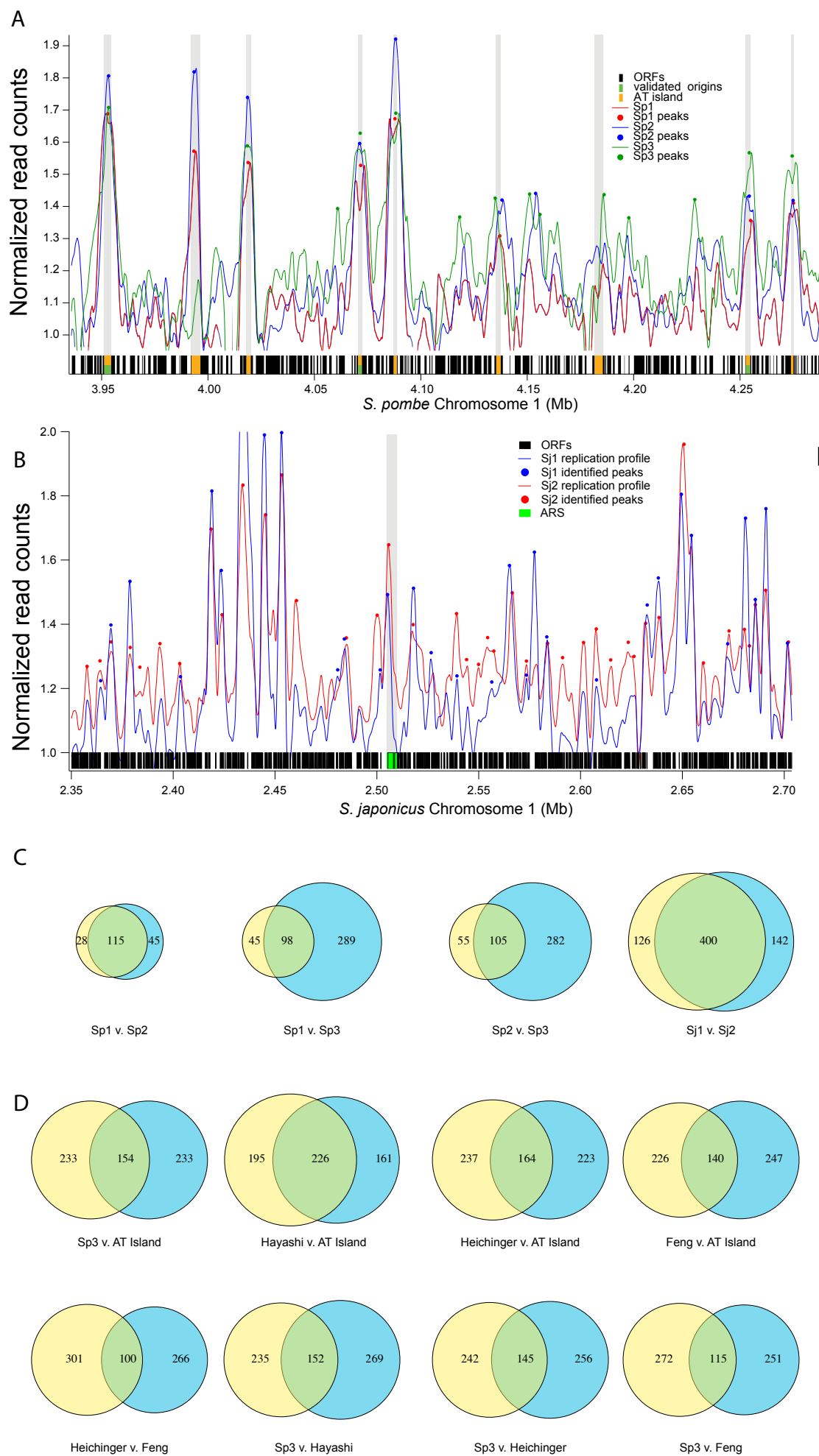

**A**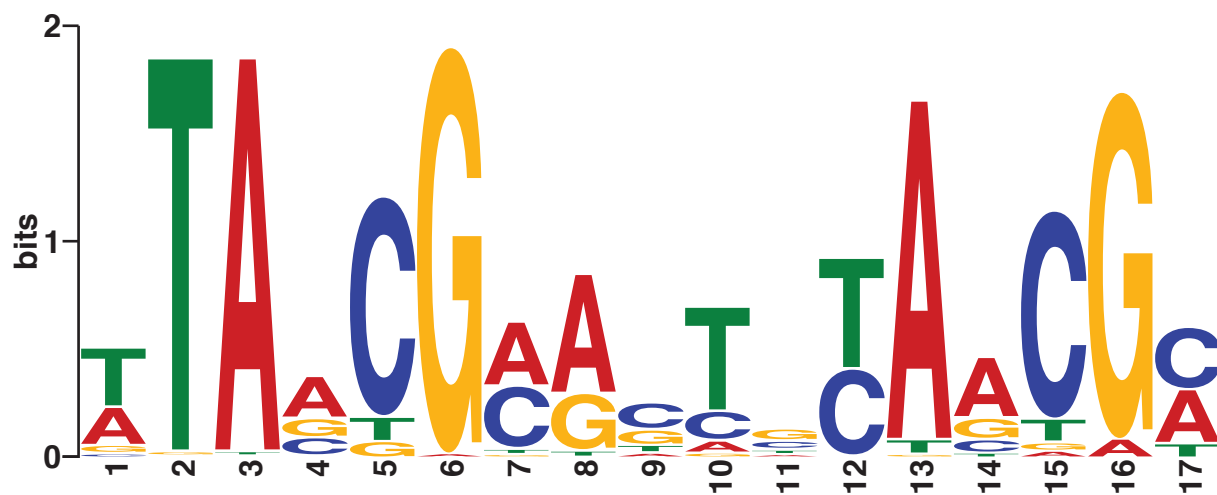**B**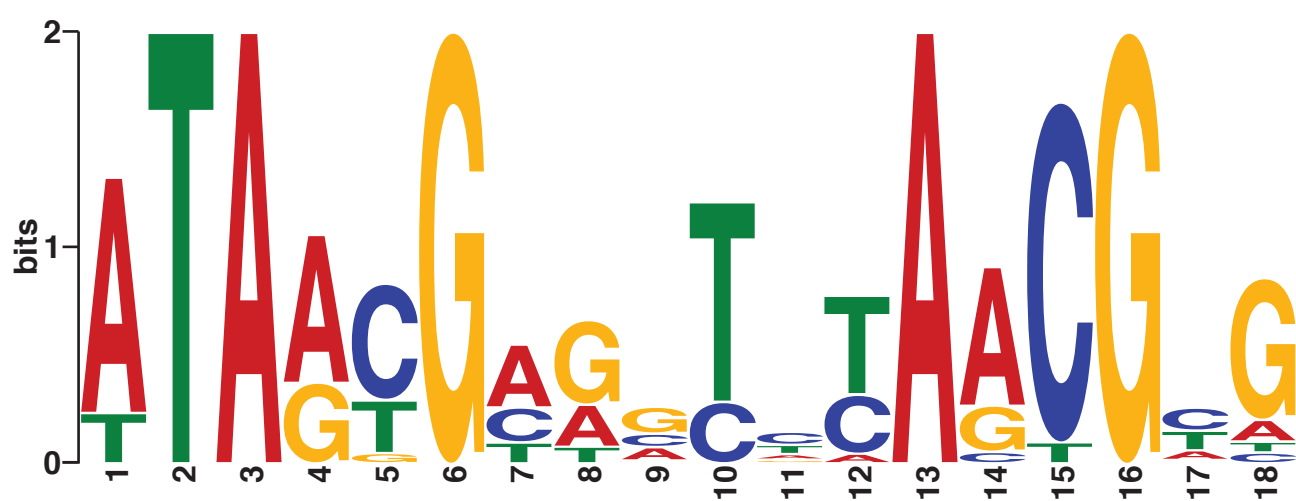

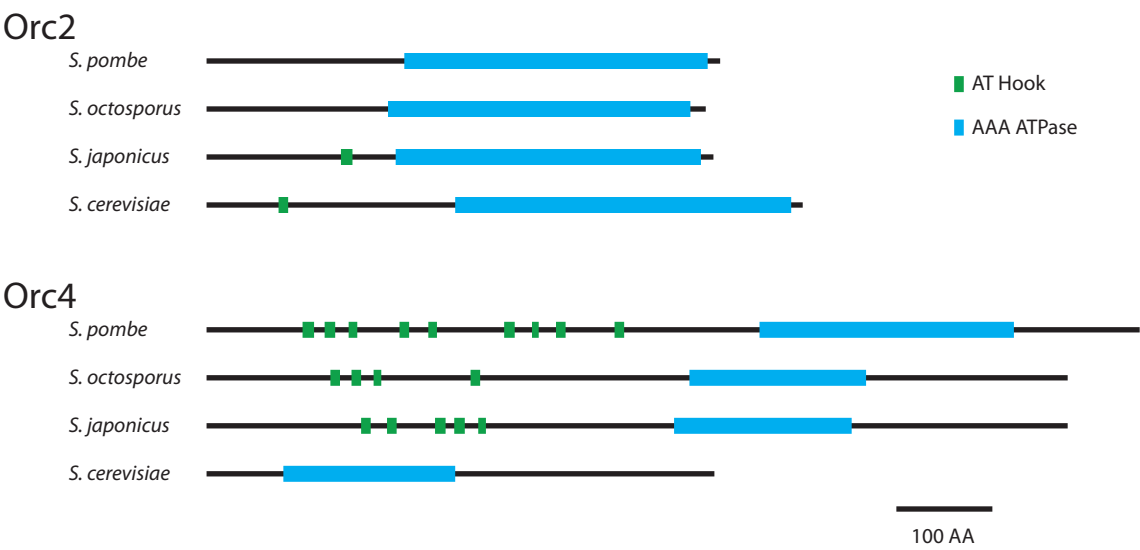

Figure S5

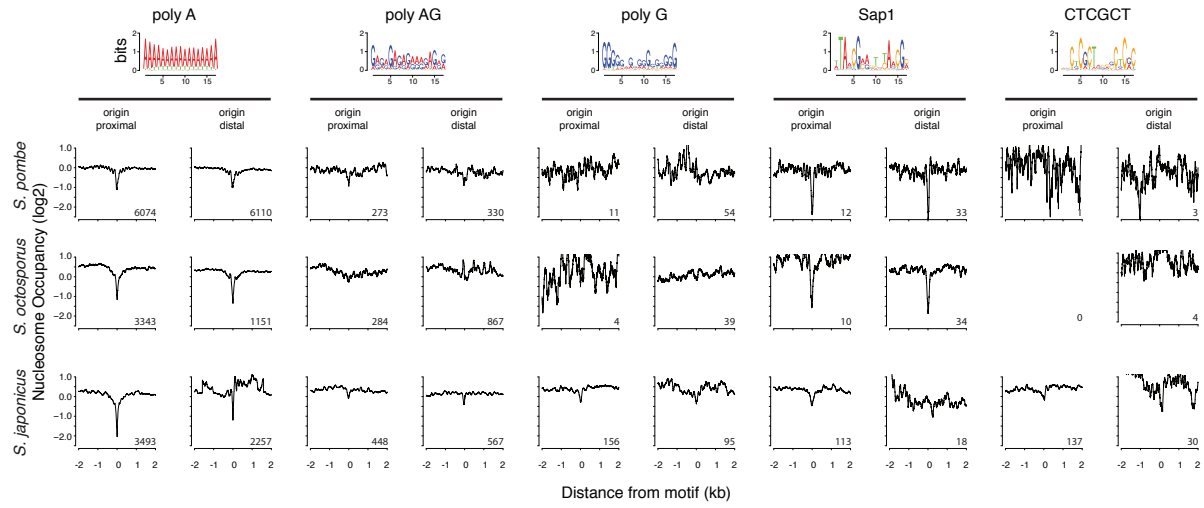

Figure S6

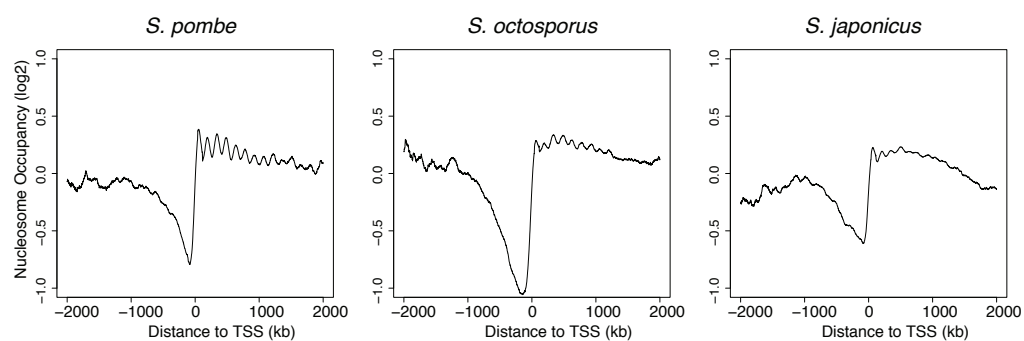

Supplement: Additional file 1 — Supplemental Figures S1 to S6. Figure S1: Peak detection by template fitting. Examples of the template-fitting approach to peak detection. The blue curve is the normalized, smoothed data. The blue dot is the peak call before template fitting. The red curve is the template. The red dot is the peak call after template fitting. The numbers are serial numbers assigned to the peaks during the iterative peak-calling process. Peak 106 can be seen at about 4.27 Mb in Figure 1 and Figure S2 in Additional file 1. Figure S2: Comparison of independent replication profiles. Replication profiles from (a) Sp1, Sp2 and Sp3 and (b) Sj1 and Sj2 are compared as in Figure 2. (c) Venn diagrams of peak overlap between the indicated datasets. Most cases of non-overlapping peak calls are due to the peak in one of the datasets being below the cutoff, such as at 4.23 Mb is S. pombe and 1.55 Mb is S. japonicus. Figure S3: The Sap1 binding site. Logos for the Sap1 binding site derived from (a) MEME analysis of S. japonicus origins (Figure 3; Table S6 in Additional file 2) and (b) in vitro selection [54]. Figure S4: Orc2 and Orc4 domain structures. Domain structures of Orc2 and Orc4 as defined by PFAM [55]. Figure S5: Origin motifs are nucleosome depleted are origin and non-origin sites. Nucleosome occupancy over motifs is depicted as in Figure 4, except motifs are divided into those within 1 kb of a replication peak and those farther away. Figure S6: Nucleosome alignment on transcriptional start sites. Nucleosome occupancy over all annotated transcriptional start sites is depicted. [file gb-2012-13-4-r27-S1.PDF]
